# Comparison of Bioelectrical Impedance and Navy Seal Formula to Measure Body Composition in Medical Students

**DOI:** 10.7759/cureus.4723

**Published:** 2019-05-22

**Authors:** Abida Shaheen, Nismat Javed, Fahad Azam, Afrose Liaquat, Moosa Khan, Syed Mahboob Alam, Sana Mumtaz

**Affiliations:** 1 Pharmacology and Therapeutics, Shifa College of Medicine, Shifa Tameer-E-Millat University, Islamabad, PAK; 2 Medicine, Shifa International Hospital, Shifa Tameer-E-Millat University, Islamabad, PAK; 3 Biochemistry, Shifa College of Medicine, Shifa Tameer-E-Millat University, Islamabad, PAK; 4 Pharmacology and Therapeutics, Jinnah Postgraduate Medical Center, Karachi, PAK; 5 Pharmacology and Therapeutics, Shaheed Zulfiqa, Islamabad, PAK

**Keywords:** bioelectrical impedance, navy seal formula, body fat ratio, muscle mass, body mass index

## Abstract

Objectives

There are many different ways to measure body composition and bioelectric impedance is one of the most popular methods to measure body ratios. The navy-seal formula is another simple way of measuring body fat ratio which takes into account simple variables such as gender, weight, height, waist, hip and neck circumference. The objective of our study was to compare the results of body fat composition by these two methods.

Materials and methods

Height and weight were measured in 85 study participants using a wall-mounted stadiometer and digital scale. Body composition measurements were recorded using a simple measuring tape. Participants were then asked to stand on the electrical impedance machine to determine the body fat and muscle mass. Data were analyzed on IBM's statistical package for the social sciences (SPSS) version 23 (IBM, Armonk, NY).

Results

The Navy-seal formula had slightly higher values for both muscle mass and body fat ratio in both genders and across all body mass index (BMI) categories. Body fat ratio and muscle mass of both genders were similar in underweight, normal, over weight and obese participants. In males, the results on two instruments showed more similarity with the increase in BMI, whereas, in females, the results of the two methods were more similar in the normal weight category.

Conclusion

Navy-seal formula and bioelectrical impedance are both simple and reliable instruments to measure body composition in adults. The navy-seal formula can be used to screen individuals with high-fat body fat ratio whereas bioelectric impedance can be used to measure the body composition for personal monitoring.

## Introduction

There has recently been a gradual rise in the global prevalence of obesity and many associated health problems such as hypertension insulin resistance, and type 2 diabetes mellitus and dyslipidemia [1-5]. Body weight alone is not a very good indicator of health as its measurement cannot distinguish between the weight of body fat and weight of lean body mass or muscle. Even if the overall weight is within normal ranges, high body ratio can still increase the risk of insulin resistance, central obesity, elevated triglyceride levels, elevated blood pressure, proinflammatory and prothrombin state [6-7].

Body fat can be measured by different means such as skin calipers, bioelectric impedance, hydrostatic weighing, Air-Displacement Plethysmography and Dual Energy X-Ray Absorptiometry (DEXA). Most of these methods are quite costly and time consuming so there is a need to develop a simpler way to assess body fat ratios.

One of the simplest methods to measure body fat percentage is using bioelectrical impedance analysis (BIA), which underpins the principle of opposing electric current through different body tissues [8]. Body composition, the measurement of body fat in relation to lean body mass is calculated from the difference in conductivity as fat-free body mass offers minimal impedance to electrical signals owing to a large amount of water and electrolytes whereas adipose tissue shows very low conductivity to electrical current [9].

Another simpler way of measuring body fat ratio is by using the Navy-seal formula which only requires simple variables such as age, gender, height, body weight and circumferences of waist, hip and neck. Calculation of body for males is by using the formula of 86.010 x log10 (abdomen - neck) - 70.041 x log10 (height) + 36.76 while for females it is 163.205 x log10 (waist + hip - neck) - 97.684 x log10 (height) - 78.387 [10]. This method for calculating body fat ratio is convenient, inexpensive and is very less time consuming.

With this background the aim of our study was to compare the results of the US Navy Seal formula and bioelectrical impedance methods to assess the body fat percentage in a group of medical students.

## Materials and methods

This was a cross-sectional study conducted on 85 medical students of Shifa College of Medicine in Islamabad, Pakistan, who volunteered for the study. All students gave verbal and written consent after being informed about the nature of study and measurements to be done. Students were asked to fast overnight before the day of body composition measurements and to empty bladders before 30 minutes of taking measurements. Moreover, students were also asked to refrain from strenuous physical activity one day prior to the experiment.

Digital scale and wall-mounted stadiometer were used to obtain the weight and height of each participant. Body composition measurements i.e., neck, waist and hip circumferences of all participants were recorded for the US Navy Seal formula. The data was collected by a trained operator using a measuring tape. The variables of age, gender, height, weight and hip, neck and waist circumferences were entered in the US Navy Seal Calculator to determine the percentage of body fat of each participant.

The participants were then asked to stand on the electrical impedance machine to determine the percentage of body fat of each participant. Leg-to leg bioelectrical impedance analysis (LBIA) of study participants was done by using a commercial glass diagnostic weighing machine (Beurer living glass diagnostic scale BG-42). Subjects were asked to stand vertically with bare feet on contact footpad electrodes of LBIA device. Body composition measurements such as lean mass and fat mass were recorded simultaneously by preprogrammed calculations automatically done by LBIA analyzer. All these measurements were taken using the instruments available in the college's Biochemistry lab.

The data obtained was analyzed on IBM's statistical package for the social sciences (SPSS) version 23 (IBM, Armonk, NY). Descriptive statistics were used to analyze and describe the data. Frequencies and percentages were calculated for qualitative variables like gender whereas quantitative variables like age and body fat percentages were computed in mean and standard deviation (SD).

## Results

Out of 85 study participants, there were 45 (53%) male participants and 40 (47%) female participants. The demographic data are displayed in Table 1.

**Table 1:** Demographic data BMI: Body mass index; SD: Standard deviation

	Males	Females
Age in years (mean±SD)	19.76±0.773	19.47±0.960
Height in cm (mean±SD)	175.47±6.85	160.05±5.90
Weight in pounds (mean±SD)	163.91±36.80	132.69±36.70
BMI, n (%)		
Underweight	6 (13)	9 (23)
Normal	24 (53)	14 (35)
Overweight	8 (18)	12 (30)
Obese	7 (16)	5 (12)

**Figure 1:**
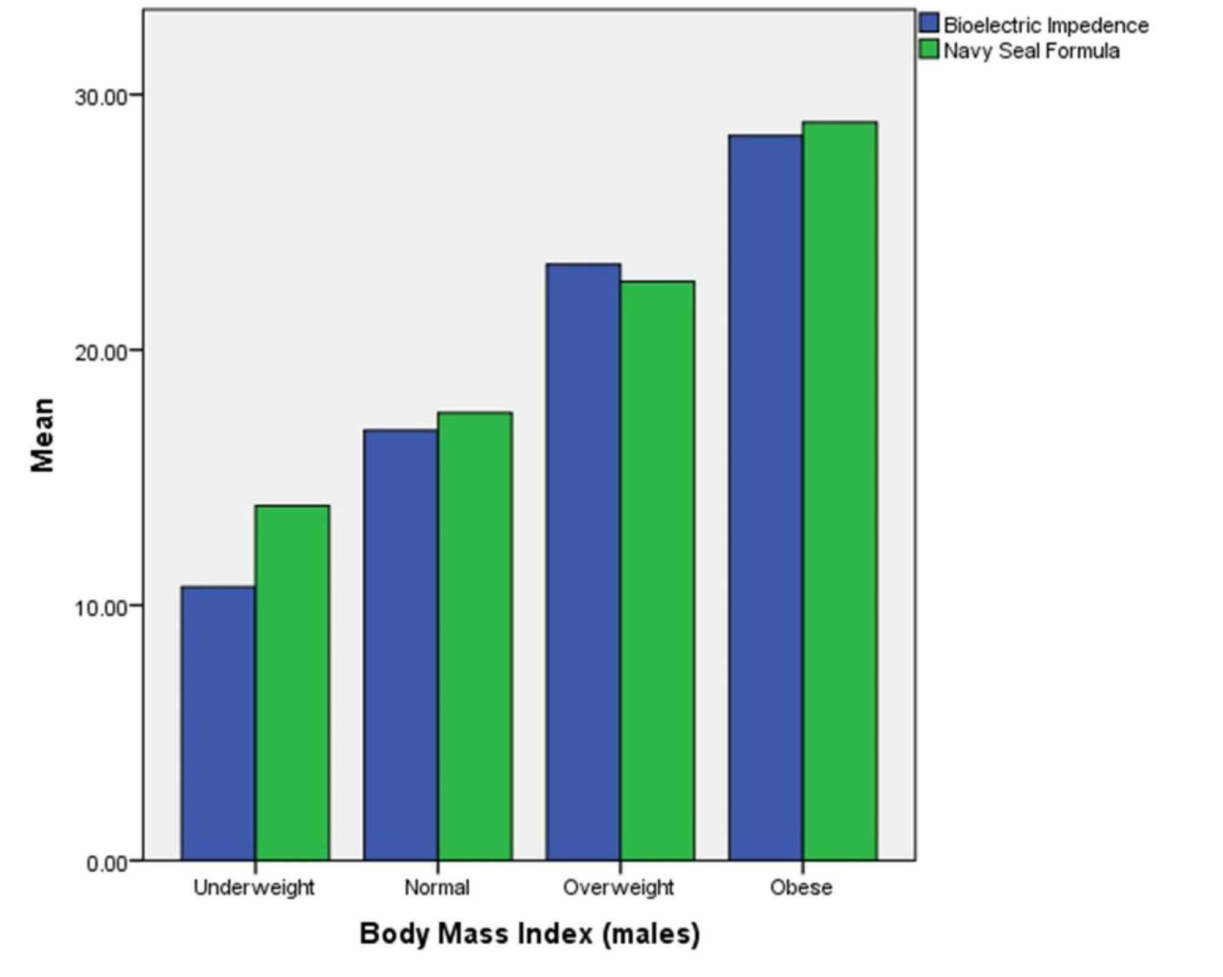
Body fat perccentage (males)

**Figure 2:**
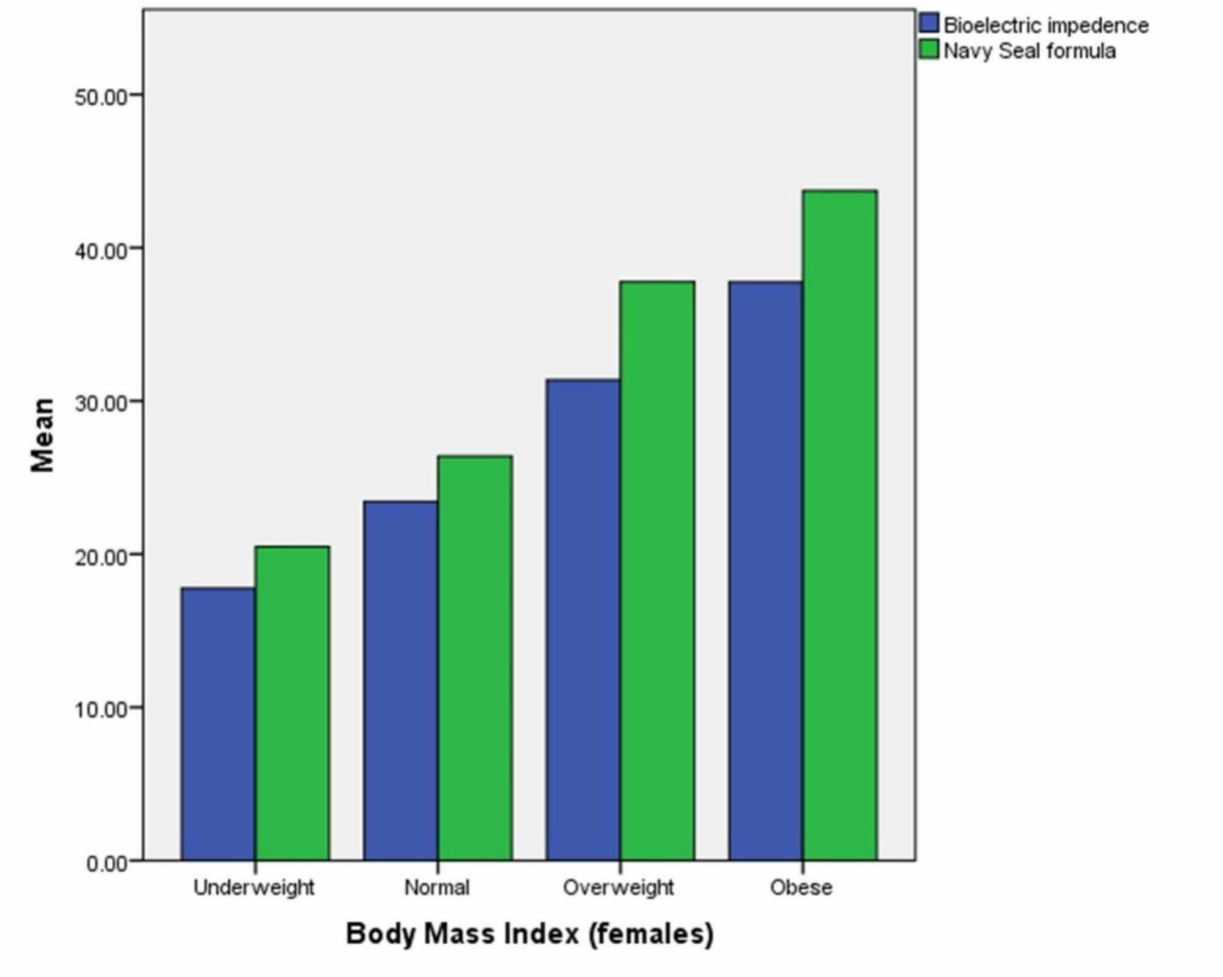
Body fat percentage (females)

The male and female participants were divided into subgroups according to their BMI. The mean values of body fat percentage recorded by bioelectric impedance and calculated by the US Navy Seal formula were plotted on a bar graph for each subgroup in each gender. The results are in Figures 1-2.

The difference in mean body fat percentage was tested using chi-square test for each gender and each subsequent subgroup and method of recording. P value less than 0.05 was considered significant. In underweight males, the p value was 0.224 when mean body fat percentage was calculated by each method while in males with a normal BMI, the p value was 0.139. In overweight males, the p value was 0.229 when mean body fat percentage was calculated by each method while in obese males, the p value was 0.227.

In underweight females, the p value was 0.220 when mean body fat percentage was calculated by each method while in females with a normal BMI, the p value was 0.241. In overweight females, the p value was 0.097 when mean body fat percentage was calculated by each method while in obese females, the p value was 0.213. 

**Figure 3:**
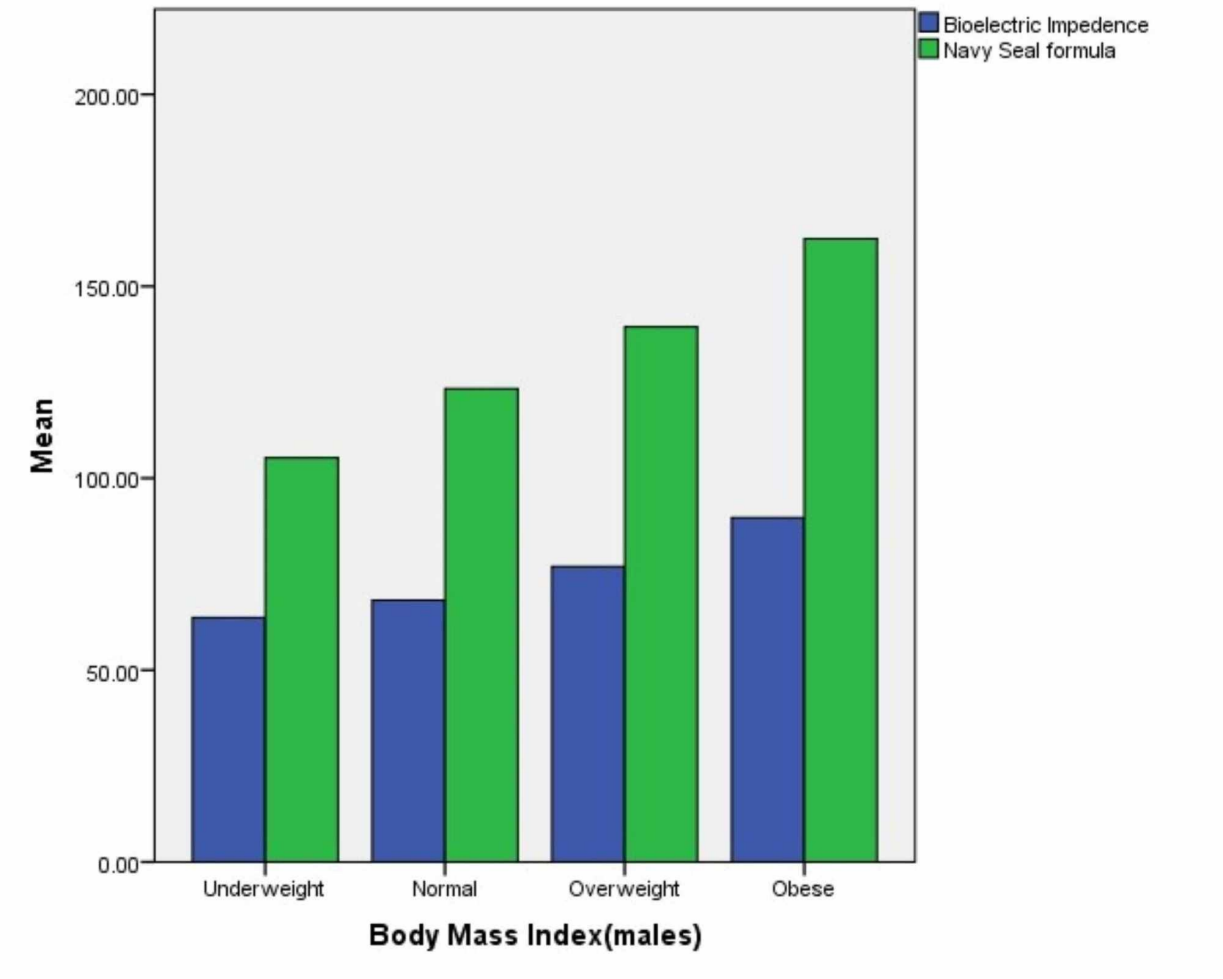
Muscle mass in pounds (males)

**Figure 4:**
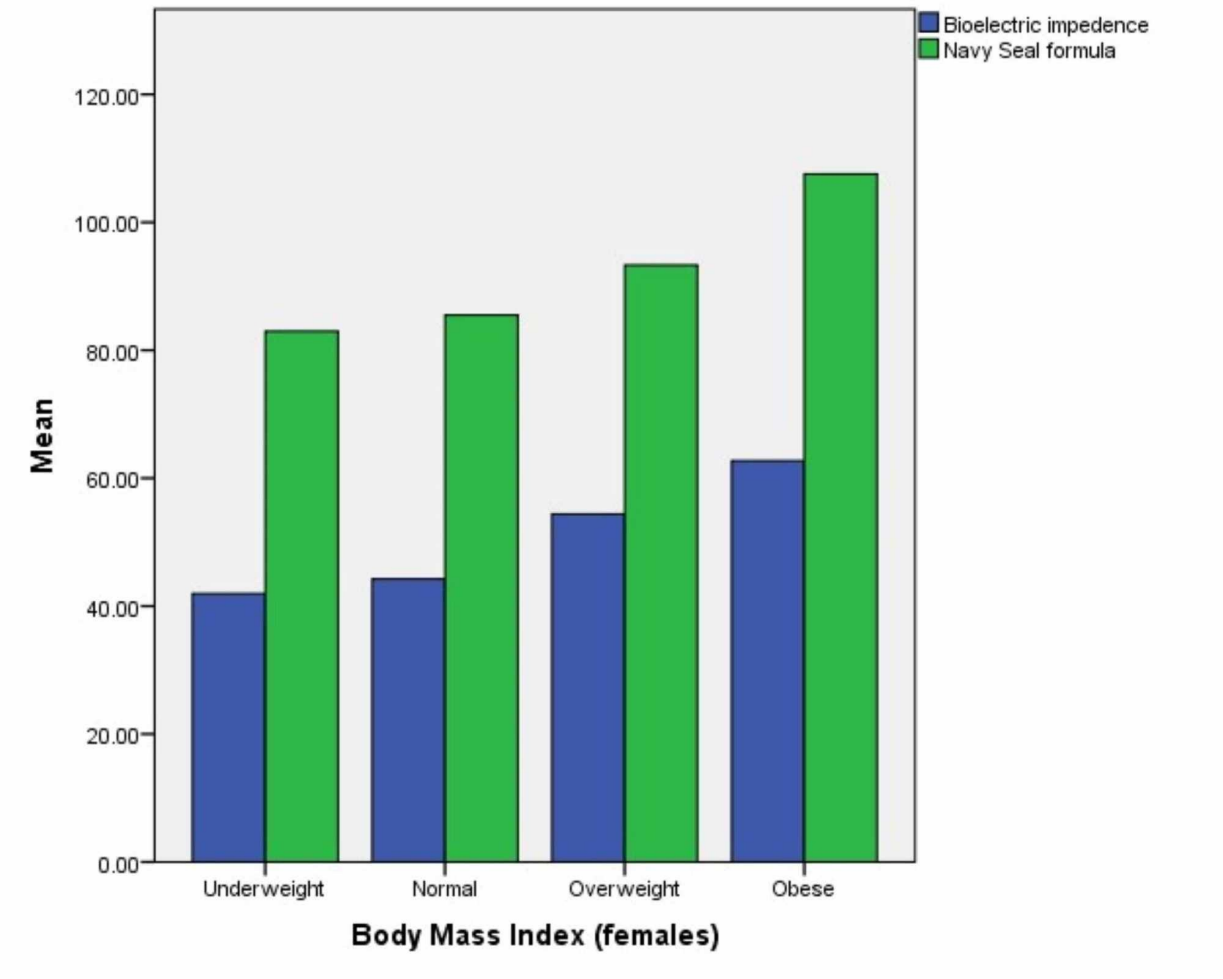
Muscle mass in pounds (females)

The mean values of muscle mass in pounds recorded by bioelectric impedance and calculated by the US Navy Seal formula were plotted on a bar graph for each subgroup in each gender. The results are in Figures 3-4.

The difference in mean muscle mass in pounds was tested using t-test for each gender and each subsequent subgroup and method of recording. P value less than 0.05 was considered significant. In underweight males, the p value was 0.224 when mean value of muscle mass was calculated by each method while in males with a normal BMI, the p value was 0.082. In overweight males, the p value was 0.229 when mean value of muscle mass was calculated by each method while in obese males, the p value was 0.227.

In underweight females, the p value was 0.220 when mean value of muscle mass was calculated by each method while in females with a normal BMI, the p value was 0.253. In overweight females, the p value was 0.083 when mean value of muscle mass was calculated by each method while in obese females, the p value was 0.213.

## Discussion

A very simple indicator of good health is BMI score and according to our results most of our study participants had normal BMI. Results of our study are in agreement with some other studies conducted in medical schools where the BMI of students was assessed [11]. Generally, medical students have a higher level of awareness about healthy diet and realize the importance of physical activities. As most of the undergraduate students are usually actively involved in sports in leisure time, therefore the majority of them usually have a normal BMI [12-13].

Considering the epidemic of obesity and associated co-morbidities worldwide, there is an increased need to monitor such changes in all age groups by inexpensive and reliable measurement tools [14-15]. The Navy-seal formula and BIA are convenient, cost-effective and readily available methods which can be used as effective prevention and treatment strategies [16-17]. 

In our study, body fat ratios of males obtained by the BIA method compared to the ratios obtained by the Navy-seal method were statistically similar in every sub category of males according to the BMI; i.e. under weight, normal, weight, over weight and obese, respectively. The p value was considerably higher in the overweight and obese participant suggesting that the reliability of the two instruments also improves with the increase in BMI.

Similarly, body fat ratios of female participants obtained by using BIA were also statistically similar when compared to the ratios obtained by the Navy-seal method in every sub category of female participants according to the BMI categories. This shows that gender did not have any effect on the reliability of the two methods to measure body fat ratios.

The results of our study are not in agreement with the results of a study conducted on Japanese children which showed that low-body-mass females have high subcutaneous fat mass as compared to low body-mass males [18]. This can be partly because of the different metabolism and genetic body composition of Japanese people as opposed to Pakistani people.

However, in contrast to the male participants, the p values of the comparisons of the body fat ratios of the female participants revealed that the highest p values were obtained in the normal BMI category.

When we compared muscle mass of males in each BMI category, we observed that muscle mass ratio was similar in all BMI categories but the highest p values were obtained in overweight and obese participants. The results were in agreement with the body fat ratio results which had the highest reliability in overweight and obese participants. Similarly, although the muscle mass ratios obtained by both methods in females were statistically similar in all categories we had the highest p values in the underweight and normal weight participants.

Overall, we observed that the Navy-seal formula had slightly higher values for both muscle mass and body fat ratio in both genders and across all BMI categories in comparison to the BIA method, however, the differences were not statistically significant. To our knowledge, this comparison between results of the Navy-seal formula with BIA method to find out body composition has not been conducted before.

A limitation of using the bioelectric impedance device is that immediately after physical activity and intake of food and water, there might be a fluctuation in the results. In contrast, the results given for body composition given by the Navy-seal formula remain constant almost throughout the day and do not fluctuate with immediate physical activity and food and water intake [19]. The Navy-seal formula could be used at any time of the day whereas a major limitation of the BIA method is that users have to use at a specific time of a day.

## Conclusions

According to results of this study, we conclude that the Navy seal formula and BIA are both quick, noninvasive and reliable instruments to measure body composition in adults and can be used as a screening tool for prevention and treatment of obesity at national level. Other measurement of body fat percentage by gold standard i.e., underwater weighing and DEXA could be difficult to perform on regular basis and costly. Moreover, the Navy-seal formula has the potential to be used as a screening tool whereas the BIA method can be used as a quick and easy method to gauge personal fitness goals.
